# Author Correction: Light and matter co-confined multi-photon lithography

**DOI:** 10.1038/s41467-024-48298-x

**Published:** 2024-05-03

**Authors:** Lingling Guan, Chun Cao, Xi Liu, Qiulan Liu, Yiwei Qiu, Xiaobing Wang, Zhenyao Yang, Huiying Lai, Qiuyuan Sun, Chenliang Ding, Dazhao Zhu, Cuifang Kuang, Xu Liu

**Affiliations:** 1 Research Center for Intelligent Chips and Devices, Zhejiang Lab, 311121 Hangzhou, China; 2 State Key Laboratory of Extreme Photonics and Instrumentation, College of Optical Science and Engineering, Zhejiang University, 310027 Hangzhou, China; 3 School of Mechanical Engineering, Hangzhou Dianzi University, 310018 Hangzhou, China; 4 ZJU-Hangzhou Global Scientific and Technological Innovation Center, 311200 Hangzhou, China

**Keywords:** Surface patterning, Polymerization mechanisms, Polymers

Correction to: *Nature Communications* 10.1038/s41467-024-46743-5, published online 16 March 2024

The original version of this Article contained an error in Fig. 2, in which the intended panel 2c was replaced with the data in panel 2e. The correct and peer-reviewed version of Fig. 2 is:


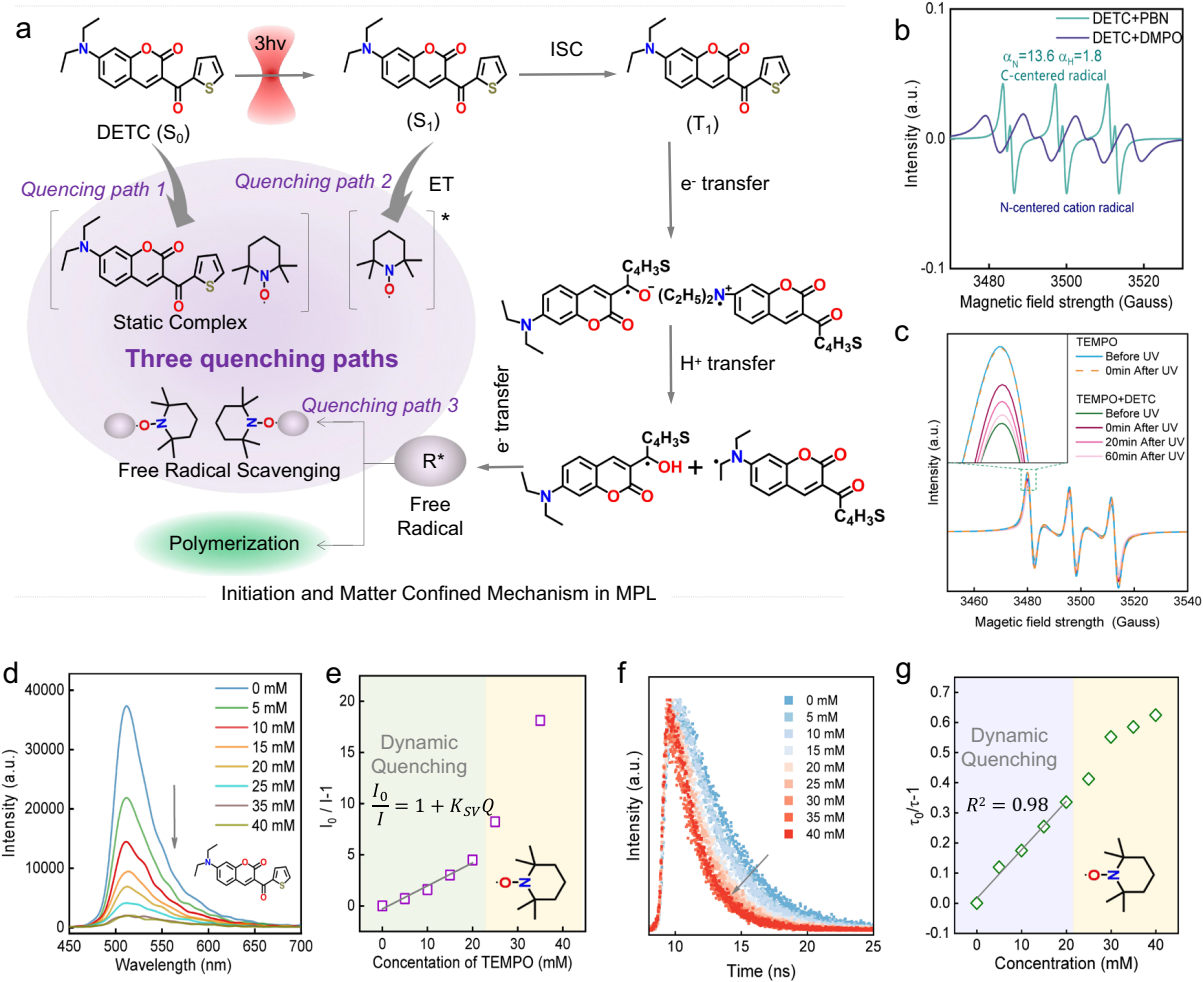


which replaces the previous incorrect version:
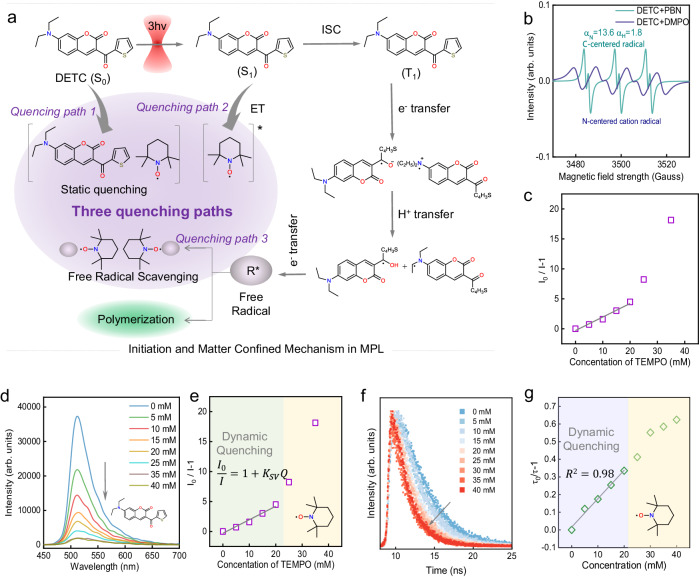


This has been corrected in both the PDF and HTML versions of the Article.

