# Supplementary material for: Light and matter co-confined multi-photon lithography
Source: Nat Commun. 2024 Mar 16;15:2387. doi: 10.1038/s41467-024-46743-5 (PMC10944545; doi:10.1038/s41467-024-46743-5)
Supplement: Supplementary file 1 — Supplementary Information [file 41467_2024_46743_MOESM1_ESM.pdf]

Supplementary Information

Light and Matter Co-confined Multi-photon Lithography

Lingling Guan,[1,#] Chun Cao,[2,3,#*] Xi Liu,[1,#] Qiulan Liu,[1] Yiwei Qiu,[1] Xiaobing Wang,[1] Zhenyao Yang,[1] Huiying Lai,[1] Qiuyuan Sun,[1] Chenliang Ding,[1] Dazhao Zhu,[1] Cuifang Kuang,[2,4*] and Xu Liu [2,4*]

[1]Research Center for Intelligent Chips and Devices, Zhejiang Lab, Hangzhou 311121, China

[2]State Key Laboratory of Extreme Photonics and Instrumentation, College of Optical Science and Engineering, Zhejiang University, Hangzhou 310027, China

[3]School of Mechanical Engineering, Hangzhou Dianzi University, Hangzhou 310018, China

[4]ZJU-Hangzhou Global Scientific and Technological Innovation Center, Hangzhou, 311200, China

[#]These authors contributed equally: Lingling Guan, Chun Cao, Xi Liu

*Corresponding authors: caochun@iccas.ac.cn, cfkuang@zju.edu.cn, liuxu@zju.edu.cn

Table of Contents

Section A. Supplementary Discussion --S3

Section B. Supplementary Figures---S7

Section C. Supplementary Tables --S26

Section D. Supplementary References---S28

Section A. Supplementary Discussion

1. The proportion of free radicals generated at T_1 and T_n state. According to Fig 2a, there are two quenching paths, quenching path 2 (dynamic quenching) and quenching path 3 (active free radical scavenging by nitroxide radicals in TEMPO), to quench free radicals when the quencher concentration is low. Therefore, the total amount of radicals reduced by the quencher is equal to the sum of the amounts of radicals quenched by quenching path 2 and quenching path 3. The change of fluorescence intensity can reflect the amounts of free radicals quenched by quenching path 2, while the amounts of free radicals quenched by quenching path 3 is proportional to the concentration of the quencher. And the total reduction of free radicals can be calculated by the changing of threshold. So, the total radical concentration, R, can be described as

$$R = R_1 + R_n \qquad (1)$$

where R_1 and R_n represents the concentration of radicals generated at T_1 and T_n state, respectively.

For quenching path 2, $R_1^{'}$, the number of quenched free radicals in S_1 state, can be calculated from the change of fluorescence intensity of DETC:

$$R_1^{'} = \frac{I_0 - I}{I_0} R_1 \qquad (2)$$

Where I_0 and I is the fluorescence intensity in the absence and presence of the quencher. Besides, the number of quenched free radicals for quenching path 3 is:

$$R_2^{'} = KRQ \qquad (3)$$

The K is the kinetic constant of termination by the quencher at the third quenching process and Q is the concentration of the quencher. When the photoresist contains the quencher, R becomes

$$R = \frac{I}{I_0} R_1 + R_n \qquad (4)$$

because the S_1-state DETC will be quenched before it transitions to MPL. Therefore, the total amount of radicals quenched by the quencher is

$$\Delta R = R_1^{'} + R_2^{'} \qquad (5)$$

Thus,

$$\Delta R = \frac{I_0 - I}{I_0} R_1 + K\left(\frac{I}{I_0} R_1 + R_n\right) Q \qquad (6)$$

Owing to $R \propto P^N$ and N=3, The total change of radical concentration can be reflected by the threshold value of photoresist. So, ΔR can be calculated by

$$\Delta R = \frac{(P_{th}^3 - P_{th0}^3)}{P_{th0}} \times R_0 \qquad (7)$$

in which P_{th0} and P_{th} is threshold of photoresist without and with the quencher. R_0 represents the concentrations of free radical without the quencher.

As shown in Supplementary Table 2, the fluorescence intensity (Fig. 2d) and threshold power values (Supplementary Fig. 17) of photoresists with 0, 5, 10, 15, and 20 mM TEMPO concentration, were given. Substituting the corresponding parameters into Equation (6), it can be calculated that R_1 and R_n account for 62% and 38% respectively.

2. Mathematical simulation of MC-MPL.[1] Before discussion, it is worth noting that our model is only valid if the following assumptions are met. Firstly, the distribution of the radicals and the quenchers keeps a non-equilibrium, quasi-stationary state around the writing spot. Secondly, it is considered the termination of the radical is controlled only by the quencher molecules for simplify the calculation.

The evolution of the concentration of radicals, $R(\mathbf{r}, t)$, and the concentration of quencher, $Q(\mathbf{r}, t)$, is described as the following equations:

$$\frac{\partial R(\mathbf{r},t)}{\partial t} = S(\mathbf{r}, t) - k_Q R(\mathbf{r}, t) Q(\mathbf{r}, t) \qquad (8)$$

$$\frac{\partial Q(\mathbf{r},t)}{\partial t} = D_Q \Delta Q(\mathbf{r}, t) - k_Q R(\mathbf{r}, t) Q(\mathbf{r}, t) \qquad (9)$$

Here, $S(\mathbf{r}, t)$ stands for the generation rate of radicals induced by excitation laser. k_Q is the kinetic constant of termination by the quencher, D_Q is the diffusion coefficient of the quencher, Δ is the Laplace operator.

For simplicity, we consider the generation rate of the radical as a spherically-symmetric Gaussian photointiation rate profile based on a Gaussian excitation beam:

$$S(\mathbf{r}, t) = S_0 \exp\left(-\frac{r^2}{2\omega^2}\right) \qquad (10)$$

Where, S_0 is the generation rate of radicals at the central spot position, $S(\mathrm{r} = 0) = S_0$. Besides, As mentioned before, the quasi-equilibrium state of the radicals illustrates that the concentration distribution of the quenchers and radicals remains unchanged during the irradiation process, that means $\partial Q/\partial t$ = 0 and $\partial R/\partial t$ = 0. Substituting the above equation into Equation (8) and Equation (9), we can get:

$$R(r) = \frac{S(r)}{k_Q Q(r)} \qquad (11)$$

$$S(r) = D_Q \Delta Q(r) \qquad (12)$$

For the initial conditions for Equation (8) and Equation (9) satisfy:

$R(\mathbf{r},0) = 0$ and $Q(\mathbf{r},0) = Q_0$.
and with the boundary condition

$$Q|_{r\to\infty} = Q_0,$$

the exact solution of Equation (11) and Equation (12) for Gaussian $S(r)$ are

$$Q = Q_0\left[1-\beta\sqrt{\frac{\pi}{2}}\frac{\omega}{r}\mathrm{erf}\left(\frac{r}{\sqrt{2}\omega}\right)\right] \qquad (13)$$

$$R = S_0\exp\left(-\frac{r^2}{2\omega^2}\right)\Big/\left\{k_Q Q_0\left[1-\beta\sqrt{\frac{\pi\omega}{2r}}\mathrm{erf}\left(\frac{r}{\sqrt{2}\omega}\right)\right]\right\} \qquad (14)$$

Where $\beta = S_0\omega^2/Q_0 D_Q$. Since it is the shape of the distribution of radicals that determines the accuracy and resolution of lithography, dimensionless variables Q/Q_0, $RK_Q Q_0/S_0$and $x = r/\omega$ are adopted to describe the Equation (13) and Equation (14). Thus, β becomes the only factor that determines the stationary solution of Equation (12). It can be seen from Equation (12) that $Q_{min} = Q(r=0)$, so

$$Q_{min} = Q_0 - S_0\omega^2/D_Q = Q_0(1-\beta) \qquad (15)$$

The dimensionless distributions of the quenchers and radicals with different values of β, are given in the Supplementary Fig. 18a and Supplementary Fig. 18b.
As the models show, there are two cases taking $\beta = 1$ as the critical point. When $\beta < 1$, the scarcity rate of the quencher in the center is slower than its diffusion rate, leading the quencher concentration at the center of the spot reaches the minimum but is not equal to 0. And the shape of the distribution of radicals become narrower with the increasing of β. While at the situation of $\beta > 1$, the scarcity rate of the quencher in the center is less than its diffusion rate is faster than its diffusion rate. So, a zero quenchers domains forms in the center. At the range of $\beta > 1$, the shape of the distribution of radicals will widen again with the value of β increasing. Therefore, it is easy to see that there exists an optimal value of β, $\beta_{optimal} = 1$, that makes the distribution of the radicals the narrowest. Because $\beta = S_0\omega^2/Q_0 D_Q$, the concentration and the diffusion coefficient of the quencher will affect the distribution of free radicals.
For the condition of MPL (without the quenchers), the concentration of radicals, $R(\mathbf{r},t)$ satisfy

$$\frac{\partial R(\mathbf{r},t)}{\partial t} = S(\mathbf{r},t) \qquad (16)$$

Considering the assumption that the radicals keep non-equilibrium, quasi-stationary, the distribution of radicals is regard as unchanged over time. Therefore, $R(x)$ satisfy the following equations for MPL and MC-MPL, respectively.

$$\text{MPL: } R(x) = S_0 \exp\left(-\frac{x^2}{2}\right) \qquad (17)$$

$$\text{MC} - \text{MPL: } R(x) = S_0 \exp\left(-\frac{x^2}{2}\right) \Big/ \varphi(x) \qquad (18)$$

$$\varphi(x) = \frac{1}{k_Q Q_0 \left[1 - \beta \sqrt{\frac{\pi 1}{2x}} \operatorname{erf}\left(\frac{x}{\sqrt{2}}\right)\right]} \qquad (19)$$

So, we can set $\varphi(x)$ as a correct factor for LMC-MPL to calculate the distribution of radicals.

Section B. Supplementary Figures

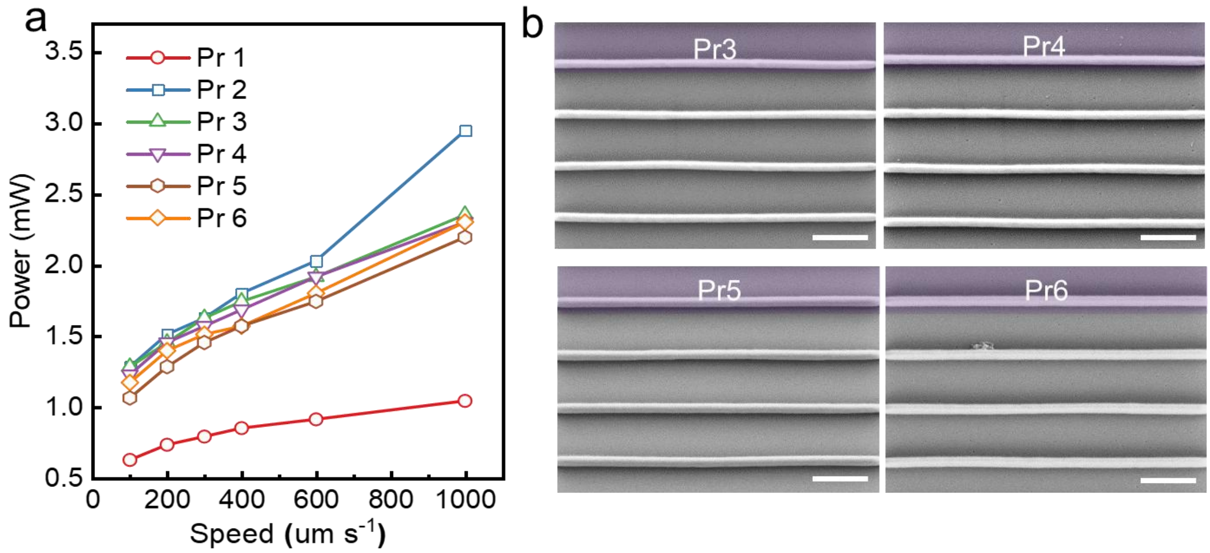

Supplementary Fig. 1 Performance of different quenchers. a The threshold power (P_{th}) curves of Pr1, Pr2, Pr2, Pr4, Pr5 and Pr6 at different writing speed. P_{th} is defined as the minimum laser power that will allow the photoresist to be retained after development. **b** SEM images of the linewidth for Pr3, Pr4, Pr5, and Pr6 (). Scale bar: 1 μm.

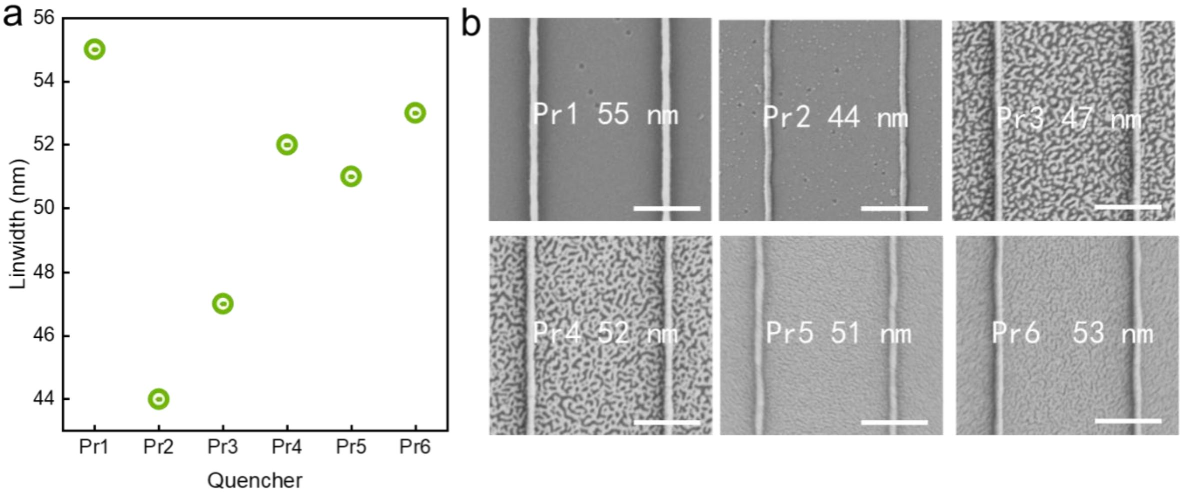

Supplementary Fig. 2 Comparison of minimum critical dimension. a Smallest linewidth of Pr1 (P_{ex}=0.58 mW), Pr2 (P_{ex}=1.10 mW), Pr3 (P_{ex}=1.05 mW), Pr4 (P_{ex}=0.98 mW), Pr5 (P_{ex}=0.87 mW), and Pr6 (P_{ex}=0.88 mW) at their threshold powers respectively (writing speed: 50 μm s^{-1}). **b** And the corresponding SEM images. Scale bar: 500 nm.

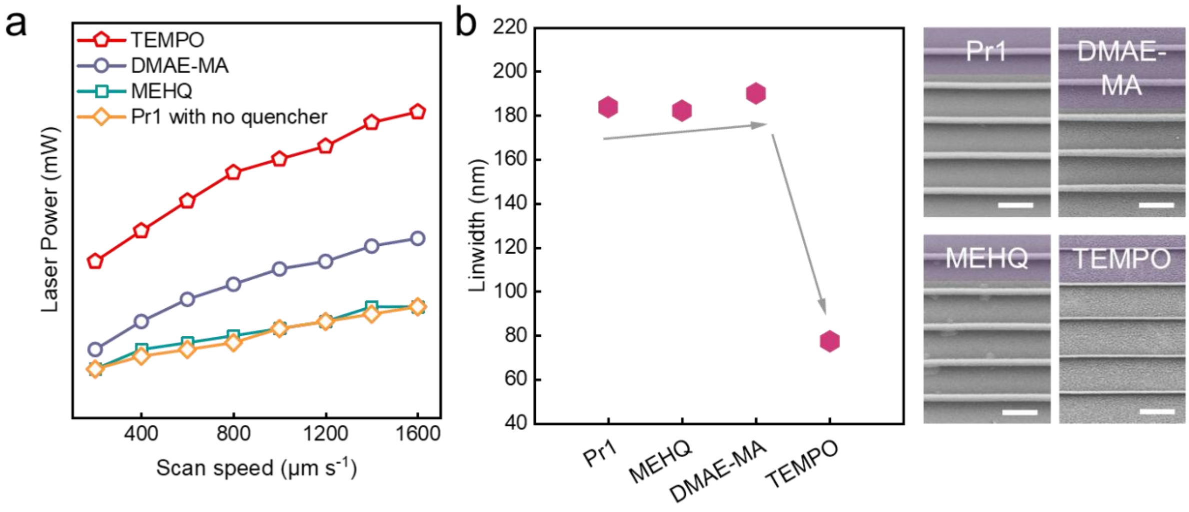

Supplementary Fig. 3 Performance of different types of quenchers. a The threshold power (P_{th}) curves of photoresists with different quenchers (TEMPO, BTPOS, DMAE-MA, MEHQ) at different writing speed. **b** SEM images of the linewidth for these photoresists at the same processing parameters (excitation beam power: 2.7 mW, writing speed: 500 μm s^{-1}). Scale bar: 1 μm.

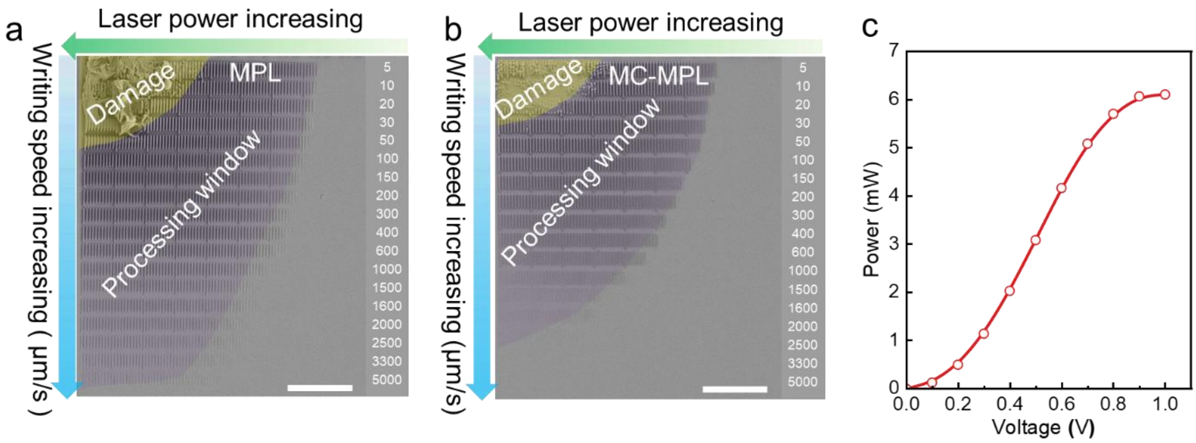

Supplementary Fig. 4 Threshold test. Threshold array of (**a**) MPL and (**b**) MC-MPL at different writing speed (5-5000 μm s^{-1}) and laser power. The laser power is controlled by the voltage of the acousto-optic modulator (from right to left: 0-1 V, Step: 0.01 V). In the damaged area, the photoresist cannot undergo controlled polymerization, but will be directly destroyed by the laser. The adjacent boundary power between the damage area and the processing window is defined as the damage power. The processing window is defined as the range of excitation laser power between the damage power and the threshold power. Scale bar: 10 μm. **c** A corresponding plot between laser power and voltage. Here the laser power in our system is adjustable, so the values of the voltage and power will not always maintain the corresponding relationship above.

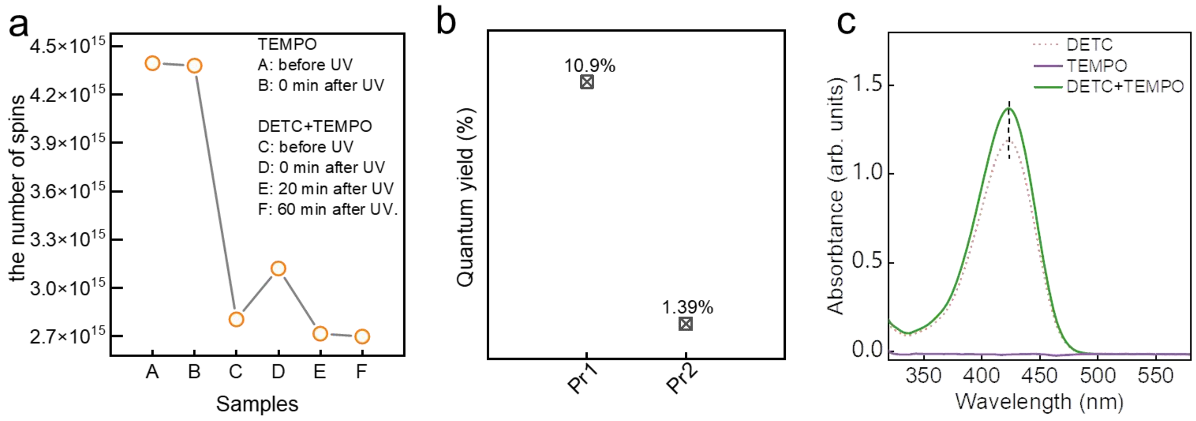

Supplementary Fig. 5 ESR results and photo-physical characterization. a The number of electron spins obtained by double integration of the ESR spectrum in Fig. 2c. **b** Fluorescence quantum efficiency of Pr1 and Pr2. **c** UV-vis absorption spectra of DETC (3.1×10^{-7} M), TEMPO (4.1×10^{-7} M) and DETC (3.1×10^{-7} M) +TEMPO (4.1×10^{-7} M). The tested substances for fluorescence quantum efficiency and UV-vis absorption spectra were dissolved in the monomer of the photoresist (87.5 wt% TCDA + 12.5 wt% EBPFDA- OPPEA) for testing, and the ratio of DETC and TEMPO is consistent with Pr2.

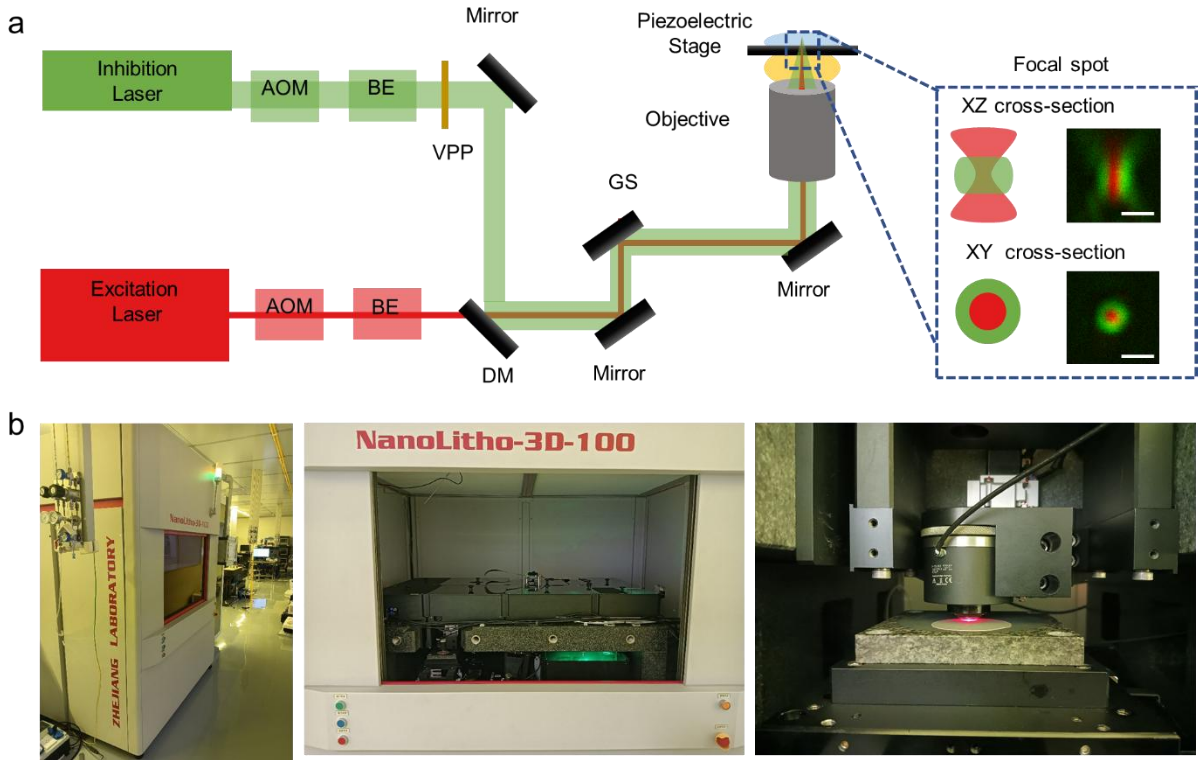

Supplementary Fig. 6 Lithography System. a A typical LC-MPL system with two beams, in which excitation beam (red) is overlapped by a shaped inhibition beam (green). AOM: acoustic optical modulator, BE: beam expander; VPP: vortex wave plate; DM: dichromic mirror; GS: galvanometer scanner; RS: rotating scanner. The schematic diagram and real picture of spots located near the x-y and x-z planes of excitation beam and inhibition beam. **b** Photos of the lithography system we used. Scale bar: 500 nm.

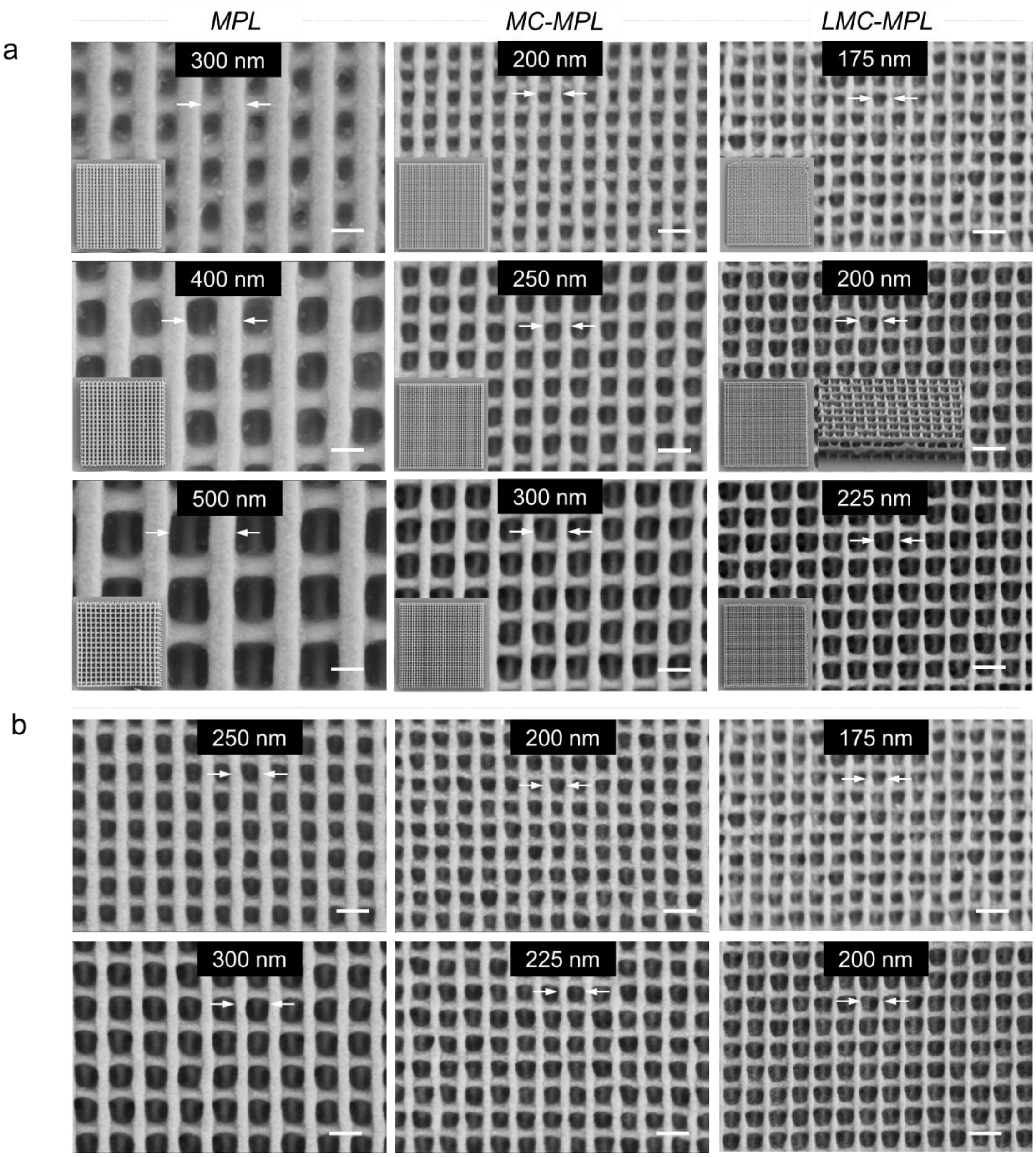

Supplementary Fig. 7 Resolution of woodpile structures. a SEM morphology of woodpile structures with different period (d) made by MPL, MC-MPL and LMC-MPL (P_{in}=9.65 mW) with fixed excitation laser power (P_{ex}=1.10 mW). **b** Woodpile structures with different period made by MPL, MC-MPL and LMC-MPL (P_{in}=9.65 mW) at their respective threshold laser power (MPL: P_{ex}=0.55 mW, MC-MPL: P_{ex}=1.04 mW, LMC-MPL: P_{ex}=1.10 mW). Writing speed=50 μm s^{-1}, Scale bar: 300 nm

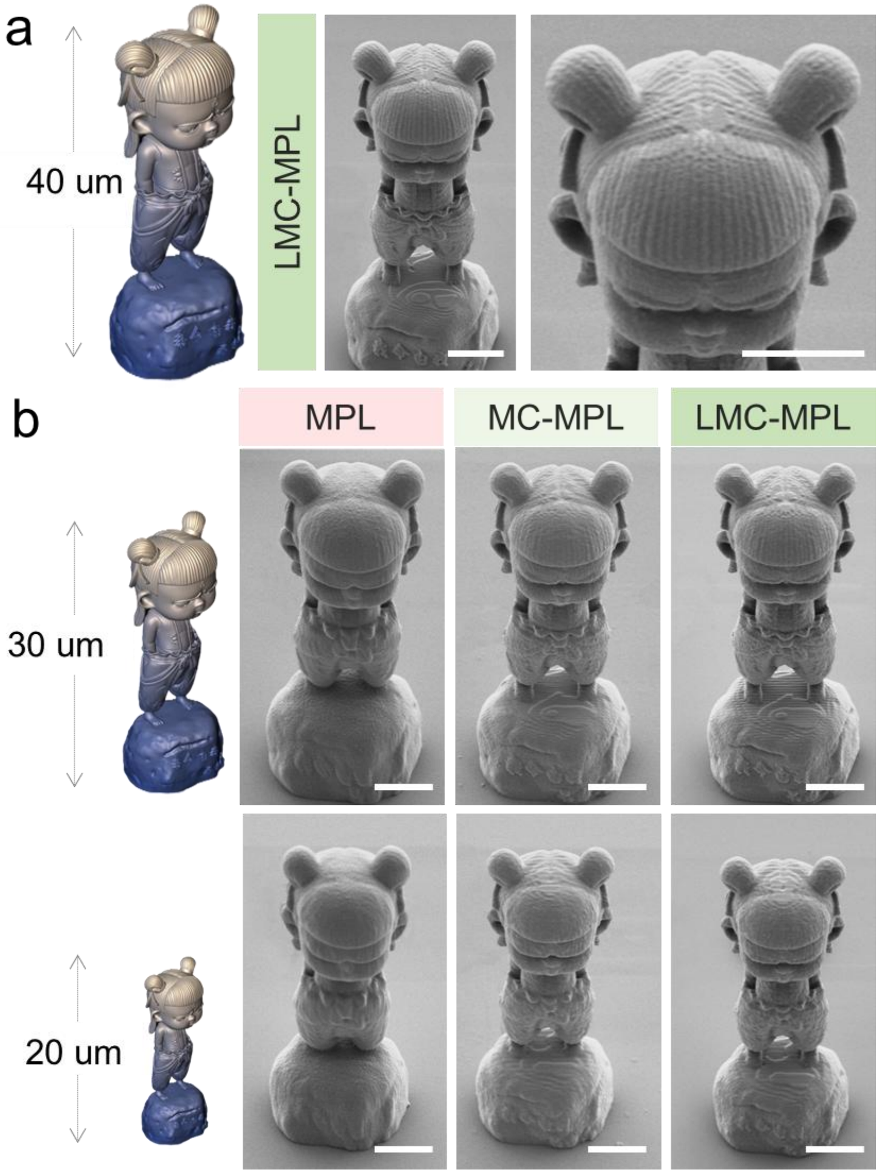

Supplementary Fig. 8 3D structure test. a SEM morphology of Nezha (40 μm in height) and the details of its head fabricated by LMC-MPL with 5.7 mW excitation beam power (P_{ex}), 9.65 mW inhibition beam power (P_{in}) and 10 mm s-1 writing speed. Scale bar: 5 μm. **b** SEM morphology of a 30 μm (Scale bar: 4 μm) and 20 μm (Scale bar: 3μm) high 3D Nezha made by different lithography modes, MPL, MC-MPL and LMC-MPL, all other processing parameters are the same (P_{ex}= 5.7 mW, P_{in}=9.65 mW and writing speed =10 mm s^{-1}).

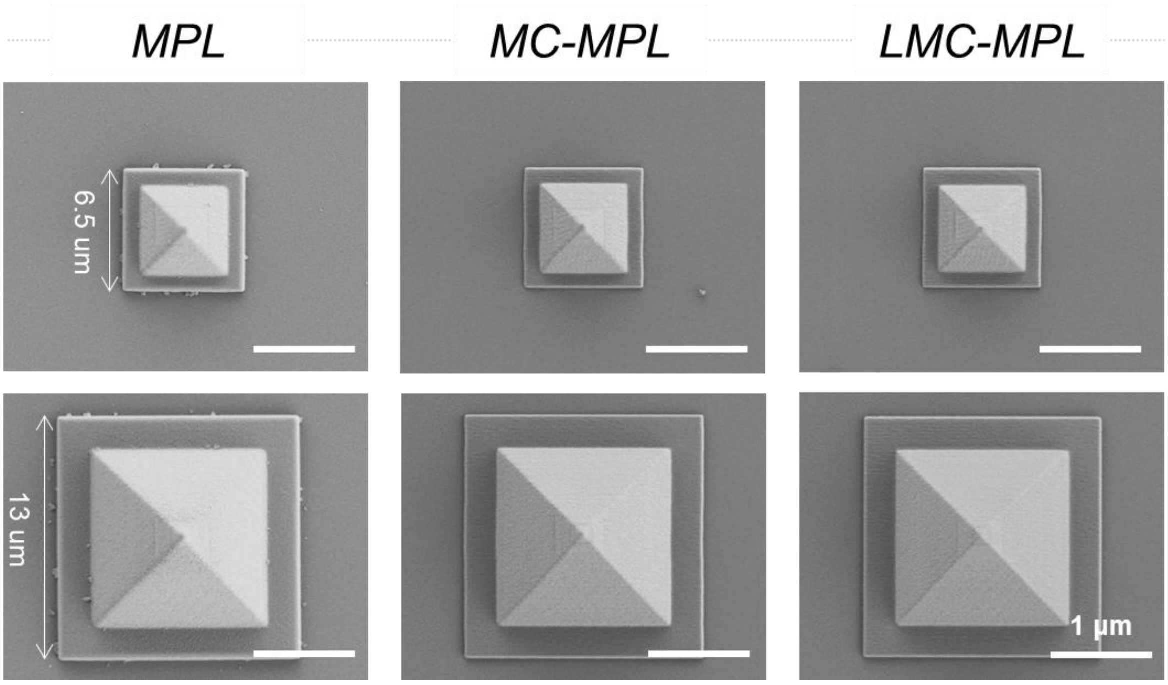

Supplementary Fig. 9 2.5D structure test. Square pyramid structures of different sizes (6.5 µm wide and 13 µm wide) with excitation beam power and writing speed fixed as 5.35 mW and 5 mm s^{-1}. Scale bar: 1 µm.

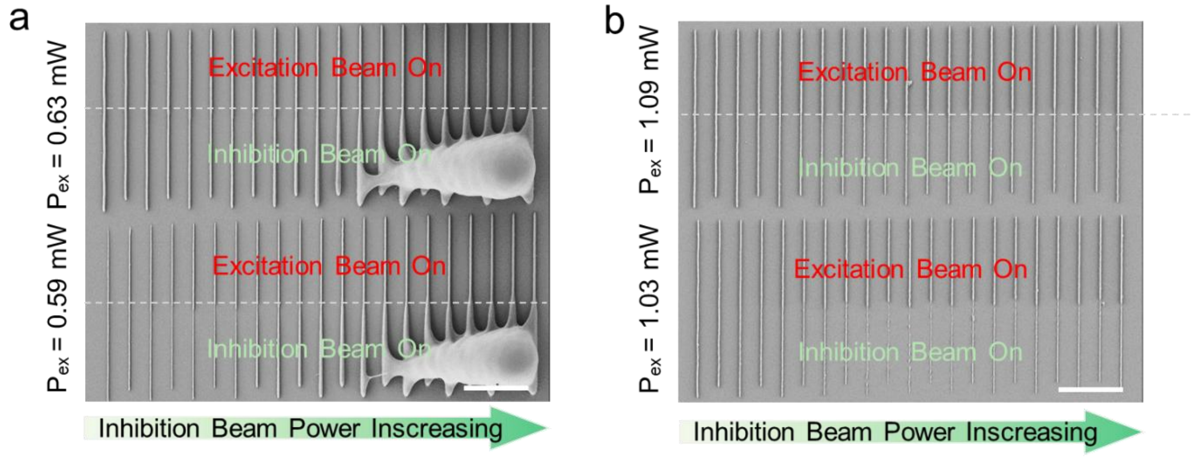

Supplementary Fig. 10 Light confining capability test patterns. SEM images of light confining capacity test patterns for (**a**) LC-MPL (Pr1) and (**b**) LMC-MPL (Pr2) with the intensity of inhibition beam increasing under fixed excitation beam power (0.59 mW, 0.63 mW for Pr1 and 1.03 mW, 1.09 mW for Pr2). Scale bar: 3 μm.

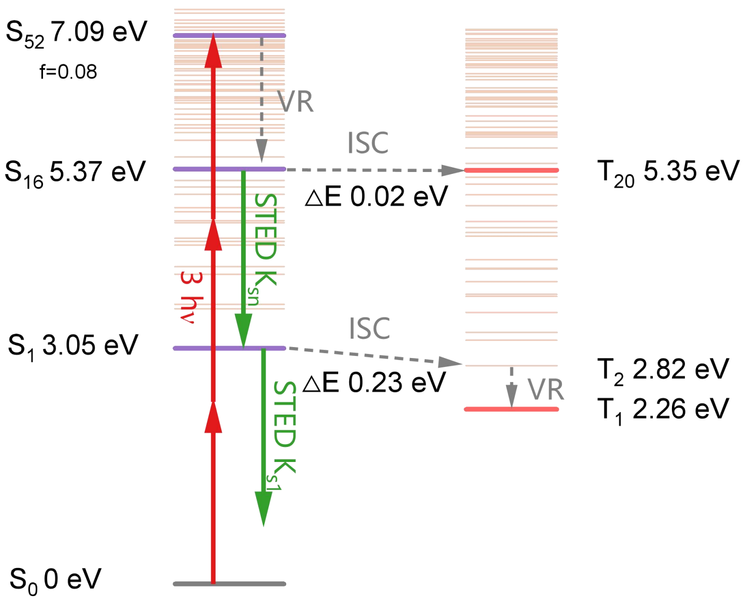

Supplementary Fig. 11 Calculated energy levels of DETC. The calculated single and triplet excited state energy level distribution of DETC by DFT (b3lyp/6-311Gd) and the possible photophysical transitions of DETC during photo-excitation.

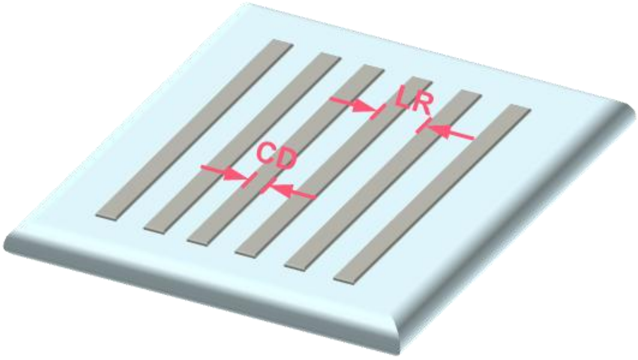

Supplementary Fig. 12 The schematic diagram of CD and LR.

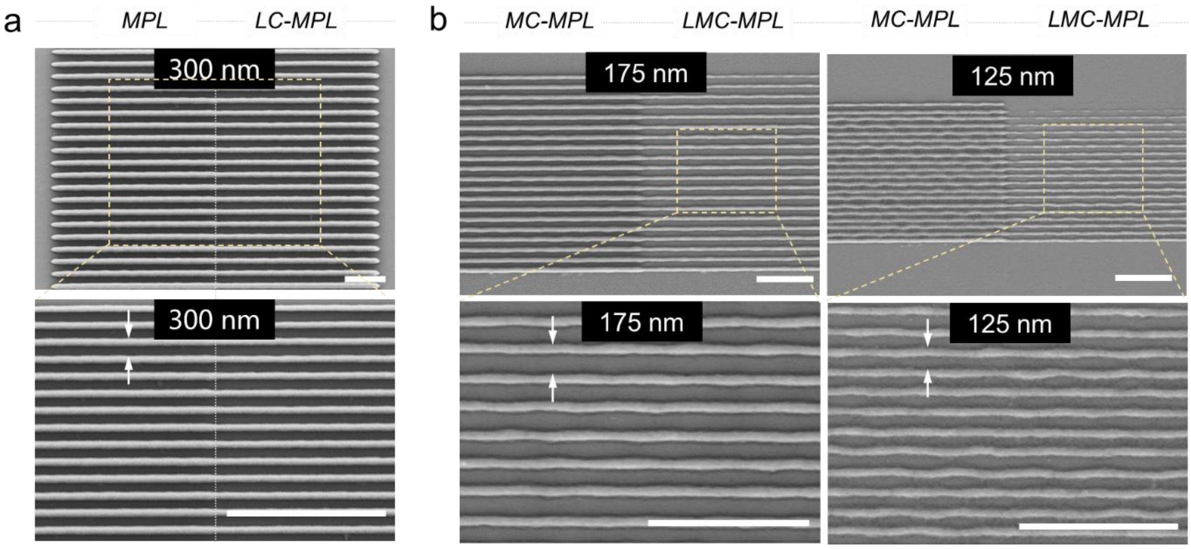

Supplementary Fig. 13 Lateral resolution test. SEM morphology of lines with different period of (**a**) MPL, LC-MPL, (**b**) (**c**)MC-MPL, and LMC-MPL with 1.05 mW excitation beam power, 9.65 mW inhibition beam power and 50 μm s^{-1} writing speed. Scale bar: 1 μm.

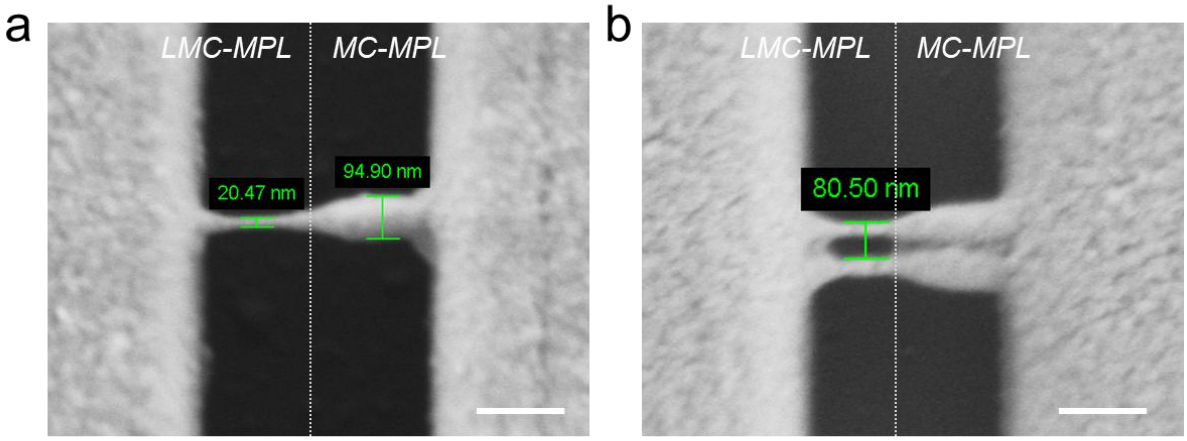

Supplementary Fig. 14 CD and LR of suspended line. The (**a**) CD (20 nm) and (**b**) LR (80 nm) of suspended line for LMC-MPL with 1.18 mW excitation beam power, 9.65 mW inhibition beam power and 50 μm s^{-1} writing speed. Scale bar: 200 nm.

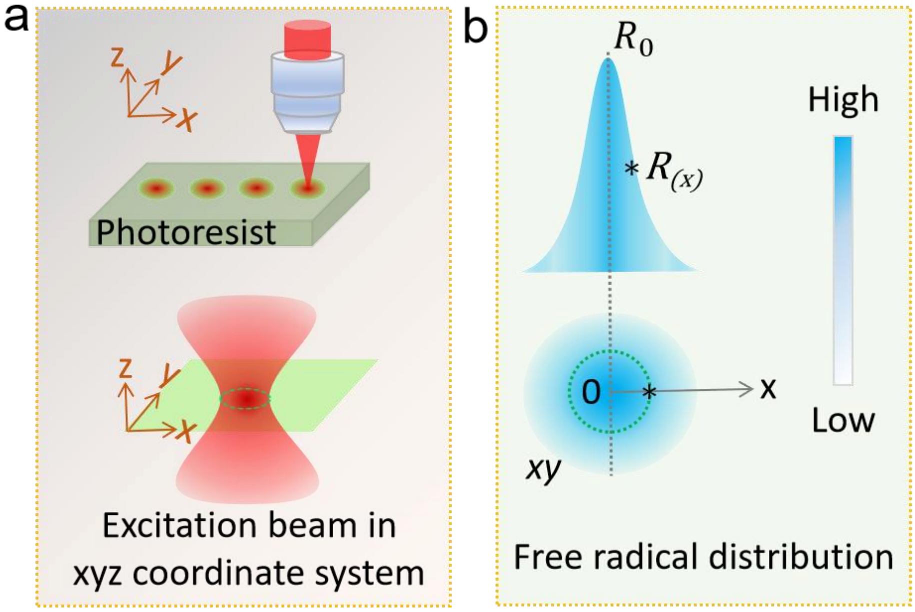

Supplementary Fig. 15 Mathematical modeling. a Three-dimensional coordinate system in MPL. **b** The distribution of free radicals in the focal beam of MPL. Where, R_0 is the free radical concentration at the center of excitation beam, and R(x) represents the concentration of free radicals at point x.

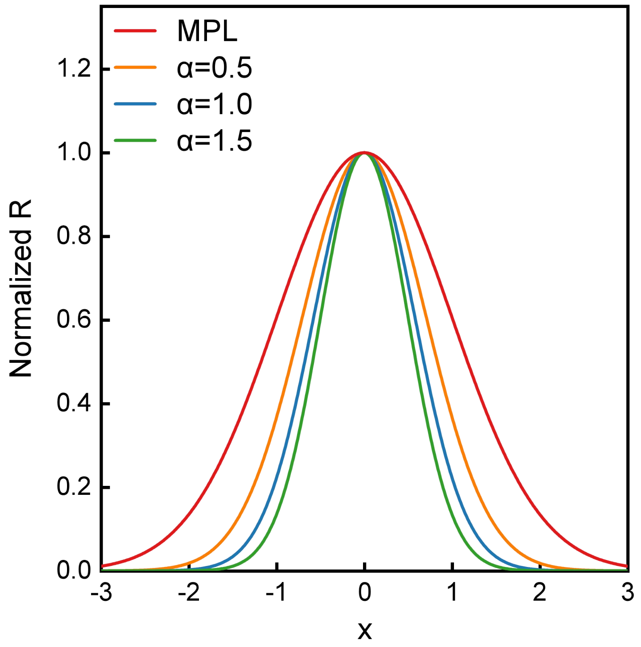

Supplementary Fig. 16 Simulation of free radical distribution. Stationary distribution of radicals for different values of α for LC-MPL compared with MPL.

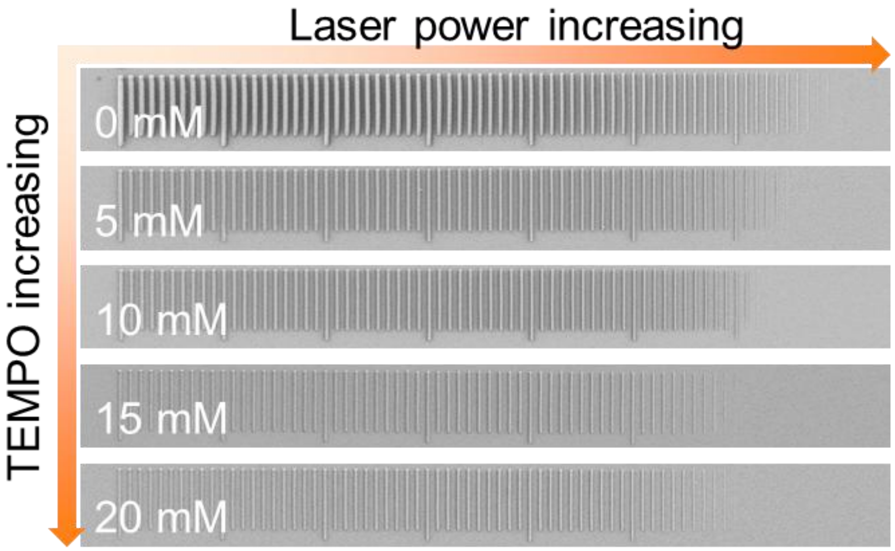

Supplementary Fig. 17 Threshold test. SEM image of threshold test at 600 μm s^{-1} scan speed for the photoresists containing different concentrations (0-20 mM) of TEMPO.

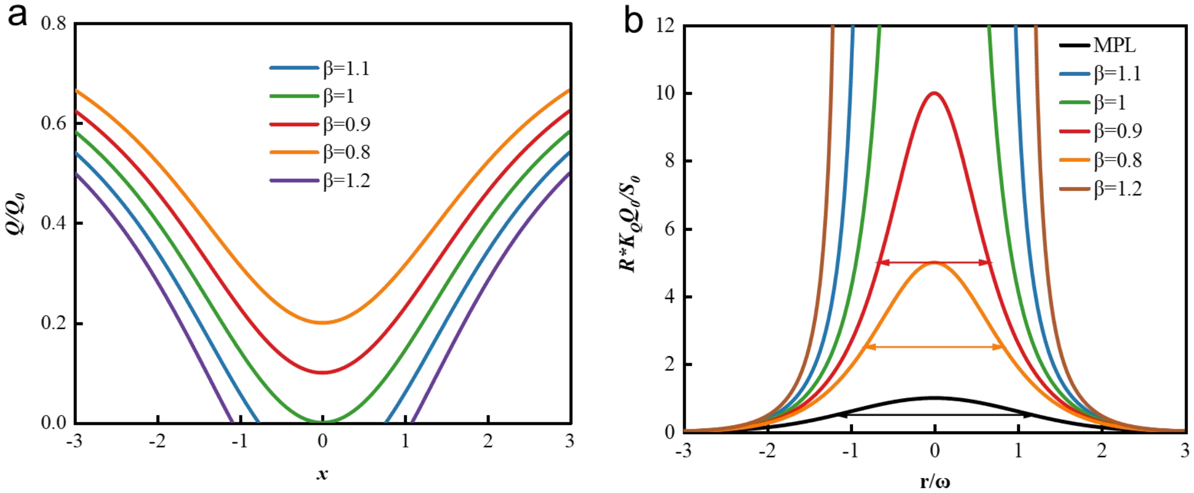

Supplementary Fig. 18 Simulation of quencher and radical distribution. a The distribution function of quenchers and **b** free radicals with different β values of MC-MPL.

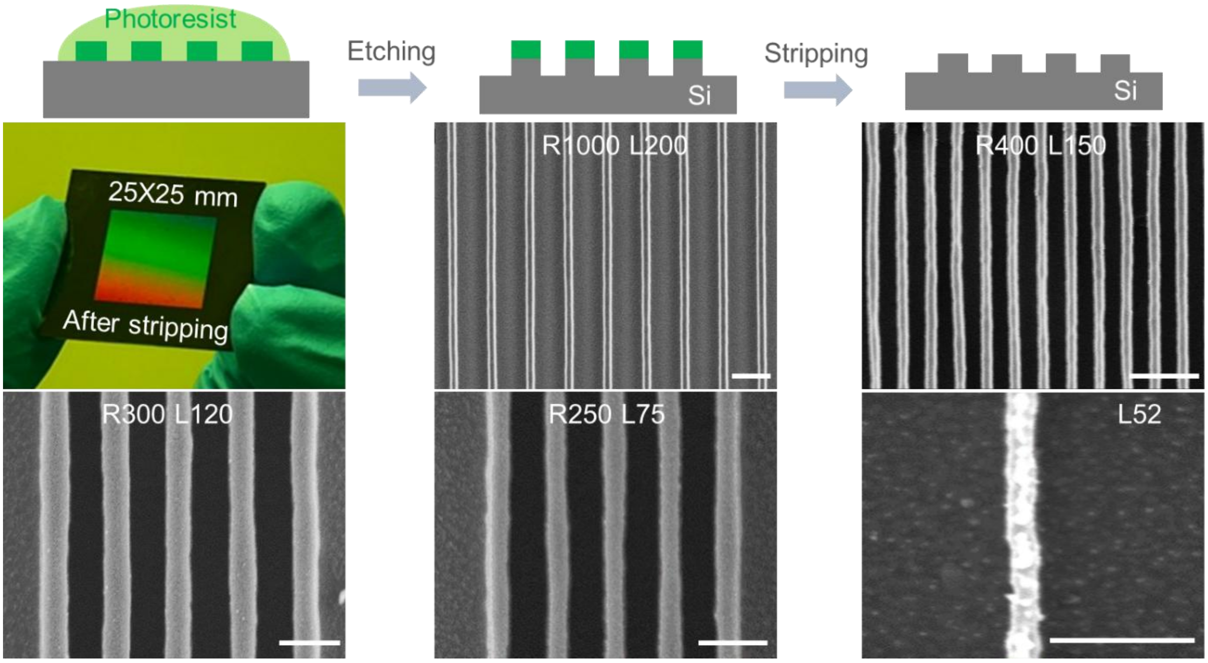

Supplementary Fig. 19 Pattern transfer. Schematic diagram of on-silicon pattern transfer process, and the corresponding optical photo and SEM photographs of the sample after stripping (R: resolution, L: linewidth). Scale bar: 1um (up) and 300 nm (down).

Section C. Supplementary Tables

Supplementary Table 1 Chemical components of various photoresists.

Samples	TCDA	OPPEA- EBPFDA	DETC	Quencher
Pr1	87.5 wt%	12.5 wt%	1 wt%	none
Pr2	87.5 wt%	12.5 wt%	1 wt%	1.28 wt% Q-1
Pr3	87.5 wt%	12.5 wt%	1 wt%	1.42 wt% Q-2
Pr4	87.5 wt%	12.5 wt%	1 wt%	1.98 wt% Q-3
Pr5	87.5 wt%	12.5 wt%	1 wt%	2.10 wt% Q-4
Pr6	87.5 wt%	12.5 wt%	1 wt%	1.50 wt% Q-5

Supplementary Table 2 Data on the proportion of radicals produced by different pathways. Fluorescence intensity (I) at 512 nm and threshold power (P_{th}) of the photoresists containing different concentrations (0-20 mM) of TEMPO

Q/mM	I/a.u.	P_{th}/mW
0	37306	0.597
5	21822	0.816
10	14395	0.912
15	9302	1.013
20	6805	1.064

Supplementary Table 3 The specific data of Fig. 4e.

Serial number	CD (nm)	LR (nm)	Lithography type	Reference
1	36	140	MPL	PhotoniX. 3, 25 (2022)
2	31	175	MPL	Chemistry of Materials. 31, 1966-1972 (2019)
3	100	175	MPL	Opt. Mater. Express. 1, 614-624 (2011)
4	55	120	MPL	Opt. Express. 21, 10831-10840 (2013)
5	32	165	MPL	Nano Letters. 21, 3915-3921 (2021)
6	100	200	MPL	Nanoscale Research Letters. 14, 134 (2019)
7	75	300	MPL	Advanced Materials Technologies. 4, 1800522 (2019)
8	150	400	MPL	Microsystems & Nanoengineering. 7, 64 (2021)
9	92	420	MPL	RSC Advances. 9, 28841-28850 (2019)
10	80	167	MPL	Microelectronic Engineering. 88, 2509-2512 (2011)
11	430	750	MPL	Cellulose. 25, 6031-6039 (2018)
12	78	120	MPL	Opt. Lett. 39, 6847-6850 (2014)
13	22	40	EUV	ACS Materials Au. 2, 343-355 (2022)
14	4	25	EBL	Nano Letters. 22, 7432-7440 (2022)
15	4.5	9	EBL	Journal of Vacuum Science & Technology B. 27, 2622-2627 (2009)

Section D. Supplementary Reference

1. A. Pikulin, N. Bityurin & V. I. Sokolov. Model of diffusion-assisted direct laser writing by means of nanopolymerization in the presence of radical quencher. AIP Advances 5, 127215 (2015).
